# High Lipoprotein(a) and Valvular and Cardiovascular Events in Patients With Aortic Stenosis: A Meta-Analysis

**DOI:** 10.1016/j.jacadv.2026.103177

**Published:** 2026-08-25

**Authors:** Arnaud S. Girard, Neil Craig, Peter E. Thomas, Marie-Ange Fleury, Børge G. Nordestgaard, Marie-Annick Clavel, Philippe Pibarot, Marc R. Dweck, Pia R. Kamstrup, Benoit J. Arsenault

**Affiliations:** a Centre de recherche de l’Institut universitaire de cardiologie et de pneumologie de Québec – Université Laval, Québec, Canada; b BHF Centre for Cardiovascular Science, University of Edinburgh, Edinburgh, United Kingdom; c Edinburgh Imaging Facility QMRI, University of Edinburgh, Edinburgh, United Kingdom; d Department of Clinical Biochemistry, Copenhagen University Hospital-Herlev and Gentofte, Herlev, Denmark; e The Copenhagen General Population Study, Copenhagen University Hospital-Herlev and Gentofte, Herlev, Denmark; f Department of Clinical Medicine, Faculty of Health and Medical Sciences, University of Copenhagen, Copenhagen, Denmark; g The Copenhagen City Heart Study, Copenhagen University Hospital-Bispebjerg and Frederiksberg, Frederiksberg, Denmark; h Department of Medicine, Faculty of Medicine, Université Laval, Québec, Québec, Canada

**Keywords:** aortic valve replacement, calcific aortic valve stenosis, cardiac mortality, lipoprotein(a)

**Figure:**
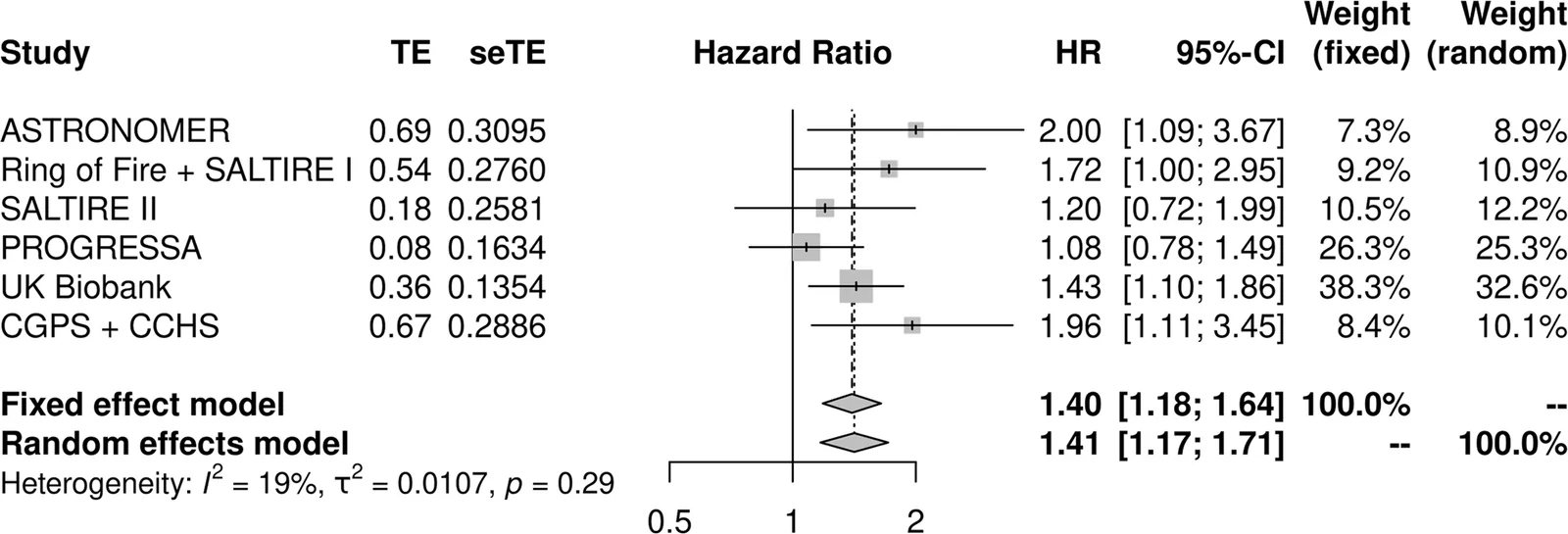

**What is the clinical question being addressed?**

Is Lp(a) associated with a higher risk of valvular and cardiovascular events in patients with aortic stenosis?

**What is the main finding?**

In patients with aortic stenosis, those with high Lp(a) levels had a higher risk of valvular and cardiovascular events.

Calcific aortic valve stenosis (CAVS) is the most common form of valvular heart disease in the Western world. Several studies over the past 15 years have reported a link between high lipoprotein(a) (Lp[a]) and the risk of CAVS.^1^, ^2^, ^3^ Lp(a) is an low-density lipoprotein-like particle that promotes inflammation and osteogenic differentiation of valvular interstitial cells.^4^ In humans, circulating levels of Lp(a) are >90% genetically determined.^1^ We recently showed that patients with CAVS and high Lp(a) levels had a faster hemodynamic CAVS progression^5^ and more risk of incidence of valvular and cardiovascular events.^3^ The objective of this study was thus to comprehensively investigate the impact of high plasma Lp(a) concentrations on the risk of valvular and cardiovascular outcomes in patients with CAVS from eight longitudinal study cohorts, including four for which such results have not been reported before.

The PROGRESSA (Metabolic Determinants of the Progression of Aortic Stenosis) study is a prospective observational cohort study that includes adult patients (mean age of 64 years) with asymptomatic nonsevere aortic stenosis (peak aortic jet velocity > 2 m/s) that included Lp(a) concentrations for 336 participants (mean Lp(a) concentration of 59.3 nmol/L). The SALTIRE II trial focused on the efficacy of denosumab and alendronic acid on disease progression for 164 patients with CAVS (mean age of 72 years and mean Lp(a) concentration of 132.1 nmol/L). The CGPS (Copenhagen General Population Study) is an ongoing cohort of individuals aged 20 to 100 years recruited from the Danish Civil Registration system. The CCHS (Copenhagen City Heart Study) includes nearly 20,000 individuals from the region surrounding Rigshospitalet, in Copenhagen, aged 20 to 100 years. Combined, the CGPS and CCHS cohorts included 260 participants with a mean age of 75 years and with CAVS (defined using the same definition as in the UK Biobank, as previously published).^3^ All studies obtained ethical approval from their respective institutional review board and all participants provided signed informed consent at recruitment. Other cohorts with previously published results regarding the incidence of aortic valve replacement (AVR) or cardiac death in patients with CAVS (Ring of Fire and SALTIRE I, n = 145,^5^ ASTRONOMER, n = 220,^5^ and the UK Biobank, n = 1,962^3^) were used to compute a meta-analysis with the results we obtained in PROGRESSA (n = 336), SALTIRE II (n = 163), and CGPS + CCHS (n = 260). The total sample size of the meta-analysis was of 3,086 patients with CAVS. The primary outcome investigated was the composite endpoint of AVR or cardiac death. In PROGRESSA and SALTIRE II, AVR was defined using the OPCS (Office of Population Censuses and Surveys) Classification of Interventions and Procedures version 4 (OPCS-4) code K26. Cardiac death was defined using International Classification of Diseases-10 codes I01-I15, I20-I25, I30-I48, I50-I51, or I70-I77. In CGPS and CCHS, AVR was defined according to the Nordic Medico-Statistical Committee (Classification of Surgical Procedures as 30780, 30810, 31268, 31269, KFMD00 to KFMD20, and KFMD96. Cardiac death was defined as death from ischemic heart disease (International Classification of Diseases-10 I20-25).

Multivariable Cox proportional hazards were used to obtain HRs and corresponding 95% CIs for the risk of the composite outcome within 5 years of follow-up associated with high Lp(a) level in PROGRESSA, SALTIRE II, and CGPS and CCHS combined. In PROGRESSA and SALTIRE II, patients with Lp(a) levels in the top tertile of the Lp(a) distribution were compared with participants with Lp(a) levels in the bottom and middle Lp(a) tertiles using a multivariable Cox proportional hazards model corrected for age and sex. In CGPS and CCHS, participants with Lp(a) levels ≥50 mg/dL were compared with those with Lp(a) levels ≤50 mg/dL using a multivariable Cox proportional hazards model corrected for age, sex, non–high-density lipoprotein cholesterol, systolic blood pressure, smoking status, and diabetes mellitus. Results from PROGRESSA, SALTIRE II, and CGPS and CCHS were then combined with previously published results from ASTRONOMER,^5^ Ring of Fire, SALTIRE I^5^, and the UK Biobank^3^ using an inverse-variance weighted meta-analysis. Analyses were completed using R statistical software (version 4.3.3) with the packages “tidyverse”, “foreign”, “data.table”, “lubridate”, “survival”, “survminer”, and “meta”. All used packages are available directly from the Comprehensive R Archive Network.

In PROGRESSA, patients in the top tertile of the Lp(a) distribution did not have a statistically increased risk of AVR or cardiac death compared to patients in the combined bottom and middle tertiles of the distribution (HR: 1.08; 95% CI: 0.78-1.49; *P* = 0.65). Similar results were obtained in the SALTIRE II trial patients (HR: 1.20; 95% CI: 0.72-1.98; *P* = 0.49). In CGPS and CCHS, participants with Lp(a) levels above 50 mg/dL were at a significantly higher risk of the composite outcome during the follow-up compared to participants with Lp(a) levels below 50 mg/dL (HR: 1.96; 95% CI: 1.11-3.44; *P* = 0.019).

The meta-analysis included 1,777 males and 1309 females with CAVS at baseline. During the follow-up, 588 had an event of AVR or cardiac death. Both models used for the computation of the meta-analysis found a significant effect of high Lp(a) levels on the risk of valvular or cardiovascular outcomes. The fixed effects model found a proportional HR of 1.40 (95% CI: 1.18 to 1.64) high Lp(a) and low Lp(a) patients, whereas the random effects model found a HR of 1.41 (95% CI: 1.17-1.71). The I^2^ value of 19% for heterogeneity found a small difference between the studies.

In this meta-analysis of 3,086 patients with CAVS, we found that compared to individuals with low Lp(a) levels, those with high Lp(a) levels had a 42% increased risk of a composite outcome of AVR and cardiac death (Figure 1). These results consolidate the impact of Lp(a) on CAVS incidence, hemodynamic progression, and now association with long-term valvular and cardiovascular outcomes. Heterogeneity analyses demonstrate that the reported effects are consistent across cohorts.

The present study should be interpreted in light of several limitations. First, definitions of CAVS and elevated Lp(a) were not fully harmonized across participating cohorts. In the case of high Lp(a), some studies used cohort-specific top tertiles and others absolute thresholds, which may have introduced exposure misclassification and influenced the magnitude of the observed associations. Second, the composite endpoint included AVR and cardiac death, outcomes that differ in clinical significance and incidence across cohorts, potentially affecting the interpretation of pooled effect estimates. Types of AVR were also not available. Finally, because several of the contributing cohort analyses had been previously completed and published, retrospective harmonization of exposure and outcome definitions as well as covariates across all studies was not feasible.

Results of the present meta-analysis support the notion that high Lp(a) may represent a therapeutic candidate of interest for the prevention of valvular and cardiovascular events in patients with CAVS and that an outcome-focused Lp(a)-lowering randomized clinical trial in patients with CAVS could shed some more light on the benefits of Lp(a)-lowering in these patients.

**Figure 1:**
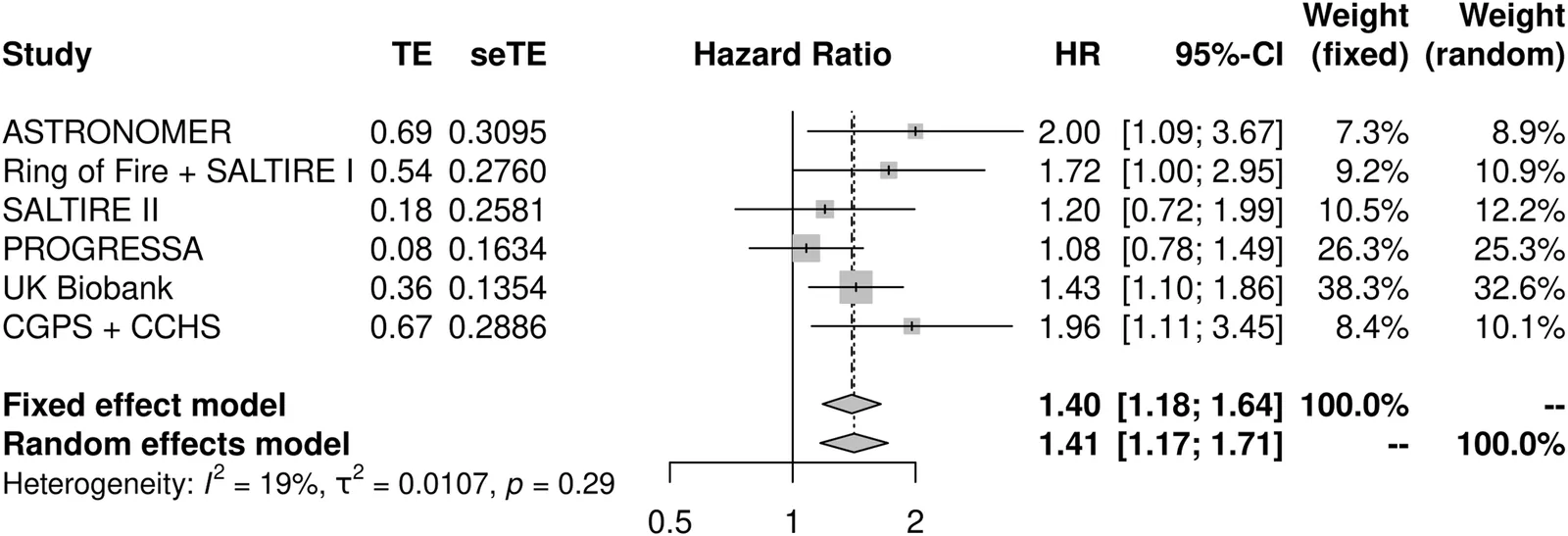
Effect of High Lipoprotein(a) in Patients With CAVS The effect of high Lp(a) levels on the risk of AVR or cardiac death in patients with calcific aortic valve stenosis (CAVS) as assessed by multivariable Cox proportional hazards model corrected for age and sex in each cohort independently. Results were meta-analyzed with inverse variance weighting using fixed and random effects models. CCHS = Copenhagen City Heart Study; CGPS = Copenhagen General Population Study; PROGRESSA = Metabolic Determinants of the Progression of Aortic Stenosis; seTE = SE of the treatment estimate; TE = treatment estimate.

## Funding support and author disclosures

Dr Arsenault has received grants from Silence Therapeutics, Eli Lilly, and Pfizer; and personal fees from Silence Therapeutics, Eli Lilly, and Novartis during the conduct of the study. Dr Craig has received grants from Medical Research Council Clinical Research Training Fellowship outside the submitted work. Dr Clavel has received grants from Edwards Lifesciences, Medtronic, and Pi-Cardia outside the submitted work. Dr Pibarot has received grants from Novartis Echo Core Lab analyses for the HORIZON CAVS trial during the conduct of the study as well as grants from Edwards Lifesciences Echo Core Lab Analyses for transcatheter valve therapies, Medtronic (preclinical in vitro analyses for TVT devices), and Pi-Cardia (Echo Core Lab analyses for TVTs) outside the submitted work. Dr Dweck has received personal fees from Novartis and Silence therapeutics during the conduct of the study. Dr Kamstrup has received lecture honoraria or consultancies from the American Heart Association, Physicians’ Academy for Cardiovascular Education, PCSK9 Forum, MedScape, Silence Therapeutics, Novartis, Amgen, and Lilly. All other authors have reported that they have no relationships relevant to the contents of this paper to disclose.
